# Alteration of protein expression and spliceosome pathway activity during Barrett’s carcinogenesis

**DOI:** 10.1007/s00535-021-01802-2

**Published:** 2021-07-05

**Authors:** Christoph Stingl, Angela Bureo Gonzalez, Coşkun Güzel, Kai Yi Nadine Phoa, Michail Doukas, Gerben Eise Breimer, Sybren Lodewijk Meijer, Jacques Johannes Bergman, Theo Marten Luider

**Affiliations:** 1 Department of Neurology, Erasmus University Medical Center, PO Box 20440, 3000 CA Rotterdam, The Netherlands; 2 Department of Gastroenterology and Hepatology, Amsterdam University Medical Centers, Amsterdam, The Netherlands; 3 Department of Pathology, Erasmus University Medical Center, Rotterdam, The Netherlands; 4 Department of Pathology, Amsterdam University Medical Centers, Amsterdam, The Netherlands; 5 Present Address: Department of Pathology, University Medical Center Utrecht, Utrecht, The Netherlands

**Keywords:** Barrett’s esophagus, Adenocarcinoma, Laser capture microdissection, Mass spectrometry, Proteomics

## Abstract

**Background:**

Barrett’s esophagus (BE) is a known precursor lesion and the strongest risk factor for esophageal adenocarcinoma (EAC), a common and lethal type of cancer. Prediction of risk, the basis for efficient intervention, is commonly solely based on histologic examination. This approach is challenged by problems such as inter-observer variability in the face of the high heterogeneity of dysplastic tissue. Molecular markers might offer an additional way to understand the carcinogenesis and improve the diagnosis—and eventually treatment. In this study, we probed significant proteomic changes during dysplastic progression from BE into EAC.

**Methods:**

During endoscopic mucosa resection, epithelial and stromal tissue samples were collected by laser capture microdissection from 10 patients with normal BE and 13 patients with high-grade dysplastic/EAC. Samples were analyzed by mass spectrometry-based proteomic analysis. Expressed proteins were determined by label-free quantitation, and gene set enrichment was used to find differentially expressed pathways. The results were validated by immunohistochemistry for two selected key proteins (MSH6 and XPO5).

**Results:**

Comparing dysplastic/EAC to non-dysplastic BE, we found in equal volumes of epithelial tissue an overall up-regulation in terms of protein abundance and diversity, and determined a set of 226 differentially expressed proteins. Significantly higher expressions of MSH6 and XPO5 were validated orthogonally and confirmed by immunohistochemistry.

**Conclusions:**

Our results demonstrate that disease-related proteomic alterations can be determined by analyzing minute amounts of cell-type-specific collected tissue. Further analysis indicated that alterations of certain pathways associated with carcinogenesis, such as micro-RNA trafficking, DNA damage repair, and spliceosome activity, exist in dysplastic/EAC.

**Supplementary Information:**

The online version contains supplementary material available at 10.1007/s00535-021-01802-2.

## Introduction

In Barrett’s esophagus (BE), the normal squamous lining of the lower esophagus is replaced by gastric type columnar epithelium [1]. This condition is considered a consequence of chronic gastroesophageal reflux disease (GERD). Because BE is asymptomatic, it is most commonly diagnosed by endoscopy in patients with GERD symptoms [2]. It is, therefore, difficult to assess the prevalence for the general population, and a biased group of patients undergo endoscopy because of symptoms that are not necessarily related to BE [3]. Dependent on the scope and population of a study, the reported average prevalence of histologically confirmed BE is around 1.5% (0.1–9.0%) [3–5]. BE is considered a premalignant precursor for esophageal adenocarcinoma (EAC), which might progress continuously through the sequence of low-grade dysplasia (LGD), high-grade dysplasia (HGD) and ultimately adenocarcinoma. It follows that both non-dysplastic BE and dysplastic BE are important risk factors for EAC [6]. The prognosis of EAC is poor; the 5-year survival rate is low, at 10–18% dependent on sex and ethnicity [7, 8]. EAC occurs predominately in males, with the highest rates in Western and Central Asia regions [9, 10], and is currently the sixth most frequent cancer, with the highest increase of incidence rate in the past 3 decades [11].

BE is diagnosed by the presence of endoscopically visible and histopathologically confirmed metaplasia [12]. The grade of dysplasia is strongly related to the risk of carcinogenesis [13] and defines the intensity of the required surveillance and treatment [14]. However, distinguishing between different grades of dysplasia is challenging and in the past resulted in low inter-observer agreement and variation in the assessment of risk of progression between studies [15]. As a consequence, the risk prediction of EAC solely on basis of the dysplastic grade is of limited reliability, potentially may lead to overtreatment [16]. The pathological progression from BE into EAC is associated with biological processes such as proliferation, tumor suppression, cell adhesion and inflammation. Molecules involved in these pathways might predict the development of EAC. A wide range of molecular markers have been studied, such as genomic alterations, epigenetic markers and proteins expression[17–19]: DNA copy number variations and aneuploidy have been found to be altered in EAC [20], and regions of loss of heterozygosity have been identified as promising predictive markers for EAC [21]. Gains of chromosomes 7 and 17 determined by FISH have been found correlated with the grade of oncogenic progression; the detection rate of dysplasia improved when the assessment of these gains was added to cytology [22]. EAC is characterized by a high mutational burden due to genomic micro-satellite instability compared to other cancers [23]. The predictive power of mutational load is limited for EAC, because in non-dysplastic BE (NDBE), the mutational load is also already elevated [24]. Correspondingly, gene expression studies have shown that the transcription profile of BE is more similar to that of EAC than that of normal esophagus [25]. Alterations of driver genes and frequency of genetic events have been found associated with EAC development [26]. Overexpression of p53, determined by immunohistochemistry (IHC), to predict development of EAC has been intensively studied [27]. Loss of heterozygosity in chromosome 17p is linked to inactivation of the p53 tumor suppressor gene. This inactivation of p53 was found in a higher frequency in HGD patients compared to NDBE patients, and is associated with a higher risk of progression to EAC [28]. Consequentially, p53 immunostaining has been suggested as an adjunct molecule marker for the diagnosis of dysplasia in BE [29].

Far fewer studies comparing the proteomes of BE and EAC tissue have been conducted. Zhao and co-workers compared premalignant Barrett metaplasia tissues with esophageal adenocarcinoma tissues taken from the same six patients. 2D liquid chromatography protein separation and time-of-flight mass spectrometry (MS) identified 38 differentially expressed proteins, of which 20 correlated with mRNA expression levels; and validated by IHC (3 of 3 proteins positive) [30]. Elsner and co-workers used imaging MS to determine *m*/*z* profiles of metaplastic and carcinogen tissue areas in a set of fresh-frozen samples taken from 38 Barrett’s adenocarcinoma patients. They found 22 *m*/*z* species that were differentially expressed and identified six of these as proteins potentially involved in tumor development and metastasis [31]. Through an LC–MS analysis of NDBE, HGD, and EAC epithelium, Zaidi and co-workers determined a diagnostic 4-protein biomarker panel that was successfully evaluated in serum by an ELISA assay on an independent cohort to discriminate between GERD and EAC patients with an accuracy of 87% [32]. O’Neill and co-workers acquired by MS-based proteomics a set of more than 6000 proteins from EAC, normal esophagus and gastric tissue samples of seven patients. Around half of the proteins quantified in tumor samples were differentially expressed, and quantification was successfully validated by IHC staining of seven proteins [33]. Despite these efforts, so far, none of the potential markers has been further developed for application in clinical practice.

In this study, we conducted an analysis specifically on the epithelial cell compartment and the surrounding stroma to determine proteomic alterations related to Barrett’s carcinogenesis. Because the proportion of epithelial cells relative to all cells of a specimen as well as the proportion of dysplastic/EAC epithelial cells relative to all epithelial cells vary widely, laser capture microdissection (LCM) was chosen as an appropriate method to collect samples that are, from a microscopical perspective, sufficiently uniform in tissue volume and stage of disease [34]. Proteins were identified and quantified by label-free bottom-up proteomics using high-resolution LC–MS. Results were validated by IHC for two selected proteins. Knowledge about these proteins and the underlying functions and pathways might add another puzzle piece to the molecular mechanisms of Barrett’s carcinogenesis. This addition could ultimately help to accurately predict the risk of carcinogenic progression, and thus decide on the most effective treatment and disease management.

## Methods

### Patient materials and characteristics

Patients were included between March 2011 and June 2015 at the Amsterdam University Medical Centers (AMC) and divided into two groups according to their histopathological diagnosis: HGD/EAC versus non-dysplastic BE. The study was approved by the medical ethics review board of the AMC (Dutch trial registration number NTR3249, https://www.trialregister.nl). Patients scheduled for EMR of BE containing HGD or early cancer were assessed for eligibility during endoscopy. Patients were excluded when the whole EMR specimen was needed for clinical decision making, when there were no visible abnormalities to target for resection, when *en bloc* resection was preferred, or when EMR was finally not performed. Eligible for inclusion in the non-dysplastic BE group were those patients with a scheduled surveillance endoscopy when no dysplasia had been found during endoscopies for at least two years previously, if no visible abnormalities in the Barrett’s esophagus had been detected in the two most recent surveillance endoscopies, and when the prior biopsies had been reviewed and diagnosed as NDBE by an expert pathologist (*N* = 11). Patients were excluded if dysplasia was found in the EMR specimen taken. For all samples used in this study, written informed consent was given from the patients prior to EMR endoscopy.

### Endoscopic mucosa resection

High-resolution endoscopy for the visualization of the Barrett segment was performed. The extent of columnar lined esophagus was documented according to the Prague C&M classification [35]. Lesions were described by the Paris classification and were resected piecemeal using the ER-cap-based technique, as described before [36]. In short, in this technique, the mucosa is lifted with saline after demarcation with coagulation. Using a transparent distal attachment placed on the tip of the endoscope, the lesion is pulled by suction into the cap. The trapped lesion is then enclosed by a snare loop and cut with electrocautery. In this study, lesions were lifted with saline without adrenaline to prevent any interaction with the proteomic analysis. We used the ER-cap technique and not the more commonly used multi-band mucosectomy technique since the latter causes venous congestion of the resection specimen which might affect proteomic analysis. Dysplastic patients were included only if the lesion was resected piecemeal, which is usually the case when the lesion has a > 2 cm diameter. The endoscopists assessed whether all specimens were needed for clinical evaluation, e.g., for the identification of the infiltration depth. If enough material was collected for clinical evaluation, the remaining material was used for this study. In the non-dysplastic group, a random portion of the Barrett segment was chosen for resection.

ER specimens were retrieved from the patient after resection and immediately pinned down, snap-frozen in liquid nitrogen, and stored at − 80 °C. Specimens for clinical evaluation were further collected according to the hospital protocol and sent for pathological review. The snap-frozen specimens were transferred on dry ice to the Erasmus University Medical Center, Rotterdam and stored at − 80 °C for proteomic analysis.

### Sample preparation

Fresh-frozen EMR specimens were cut in 8 µm thick sections that were placed on PEN membrane slides (Zeiss, Göttingen, Germany), fixated with 70% ice-cold ethanol and stored at − 80 °C until further processing. Before LCM, mounted EMR sections were thawed, hematoxylin and eosin stained, and air dried. Immediately thereafter, tissue areas of 600,000 µm^2^ were collected by LCM, yielding a tissue volume of approximately 4,800,000 µm^3^ (corresponding to an estimated number of 4800 cells under the simplified assumption that the size of a cell corresponds to a cube with an edge length of 10 µm). Micro-sections, collected in the cap of the collection tube (Zeiss *AdhesiveCap*), were then transferred using 20 µL 0.2% aqueous Rapigest (Waters, Milford, MA, USA) into a sample tube (*Eppendorf LoBind*) and stored at − 80 °C until digestion. Next, LCM microsections were thawed, heated to 95 °C for 2 min and lysed in a sonification cell disruptor (Branson sonifier, 70% intensity). Ammonium bicarbonate was added to 50 mM (final) and the tissue lysate was reduced at 5 mM dithiothreitol (1 h at 57 °C) and afterwards alkylated at 15 mM iodoacetamide (1 h in darkness at room temperature). Samples were digested by addition of 50 ng trypsin (trypsin gold, Promega, Madison, WI, USA) and over-night incubated at 37 °C. Digests were stopped and detergent (Rapigest) hydrolyzed simultaneously by addition of 0.5% trifluoroacetic acid (final), incubation at 37 °C for 1 h and subsequently centrifuged (14,000*g*, 10 min). Finally, digests were transferred to LC vials and stored at + 4 °C until measurements. Unless otherwise noted, all reagents were purchased from Sigma Aldrich.

### LC–MS acquisition

LC–MS analysis was conducted on a nano-LC system coupled to an Orbitrap Fusion mass spectrometer (Thermo Fisher Scientific, San Jose, CA, USA). Twenty µL (entire volume) of digest was loaded onto a trap column (C18 PepMap, 300 µm ID × 5 mm, 5 µm, 100 Å; Thermo Fisher Scientific) and desalted for 10 min using 0.1% trifluoroacetic acid at a flow rate of 20 µL/min. Subsequently, the trap column was switched in-line with the analytical column (PepMap C18, 75 µm ID × 500 mm, 3 µm, 100 Å) and peptides were eluted using a binary 90′ gradient increasing solvent B from 4 to 38%, whereby solvent A was 0.1% formic acid, solvent B 80% acetonitrile and 0.08% formic acid, flow rate 300 nL/min and column temperature 40 °C. For electrospray ionization, nano ESI emitters (New Objective, Woburn, MA, USA) were used and a spray voltage of 1.7 kV applied. A data-dependent acquisition MS method was used with an Orbitrap survey scan (range 375–1500 *m*/*z*, resolution of 120,000, AGC target 400,000), followed by consecutively isolation, fragmentation (HCD, 35% NCE) and detection (ion trap, AGC 10,000) of the peptide precursors detected in the survey scan until a duty cycle time of 3 s was exceeded (‘Top Speed’ method). Precursor masses that were selected once for MS/MS were excluded for subsequent fragmentation for 60 s.

Samples of each cell type and EMR specimen were prepared and analyzed in duplicate (*N* = 92 runs, of 23 samples × 2 cell types × 2 replicates). A total of 91 measurements were successfully completed (1 failed for a technical reason). The sample set was split in two parts according to the cell type (epithelial or stromal) and both sets were subsequently analyzed independently of each other. Acquired data have been made publicly available through the ProteomeXchange Consortium using the PRIDE identifier PXD020903 [37].

### Protein identification and quantification

Protein identification and label-free quantification (LFQ) was carried out, separately for epithelial and stromal samples, by the quantitative proteomics software package MaxQuant [38, 39] (version 1.6.1), using the internal search engine Andromeda [40] applying the following settings: human (*Homo Sapiens*) subset of the uniprot swissprot database (20,194 entries; version: 12. November 2015), carbamidomethylation (+ 57.021 u) of cysteine as fixed modification, oxidation (+ 15.995 u) of methionine, proline and lysine and protein N-terminal acetylation (+ 42.0106 u) as variable modification, tryptic cleavage allowing two miscleavages, 10 ppm precursor tolerance, 0.5 u fragment tolerance, and ESI-trap as instrument type. For the label-free quantification, the parameter multiplicity was set to 1, label-free quantification set to LFQ, and calculation of iBAQ values activated; otherwise the default settings were used. Next, we combined results of all samples by cell type, applied filtering of identification (local protein false discovery rate, *FDR* < 1%, local peptide *FDR* < 0.1%, minimum 2 peptide/protein identified) and conducted protein grouping using the software package Scaffold (Proteome Software, version 4.10, batch Q +). Protein-sample table containing protein abundances (iBAQ values) and spectrum reports were exported and used for further data analysis.

Statistical calculations and analysis of differentially expressed proteins were carried out with the statistical software package R [41]. First, iBAQ values of the individual runs were aggregated by sample, then ^2^log -transformed and the missing values replaced, by a sample-specific zero imputation value calculated as abundance mean minus 4 standard deviations [42]. Distributions of protein abundances were tested for normality with the Shapiro–Wilk test (normality indicated by a *P* > 0.1). Because abundances were normally distributed for just 24% of the proteins (786 of 3226), we concluded that in general the requirements for parametric tests were not fulfilled. Therefore, we used the non-parametric Wilcoxon rank-sum test in combination with Benjamini–Hochberg correction for multiple hypothesis testing to find potentially significant different protein abundances between dysplastic/EAC and non-dysplastic specimen. All proteins with an *FDR* < 5% were reported as differentially expressed proteins.

### Gene set enrichment analysis

Gene set enrichment analysis was performed on the set of differentially expressed proteins (*FDR* < 5%) queried against the protein–protein interaction (PPI) database *STRING* (https://string-db.org). This database contains pathway annotations from *KEGG* (https://www.genome.jp/kegg/) and *Reactome* (https://reactome.org/). Analysis was performed using the software *Cytoscape* (v. 3.7.2) [43]. To query the PPI network and to conduct gene set enrichment, we used the add-in Cytoscape StringApp (v 1.5.1.) [44] applying a confidence cut-off of 0.4, no additional interactors and the set of all identified and quantified proteins served as reference gene set to assess the statistical background. Prior to conducting functional enrichment, the PPI network was clustered by the interaction strength applying MCL clustering with granularity set to 2.0 using the add-in ClusterMaker [45]; subsequently functional enrichment was carried out on the four largest clusters. Pathways (*KEGG* and *Reactome*) with an *FDR* < 0.05% were exported and used for interpretation of the data. For analysis of functional similar proteins and PPI of the spliceosome-related proteins we used the software and database of *GeneMANIA* [46, 47] (through the Cytoscape App *GeneMANIA* [48], version 3.5.2; H. Sapiens data set, version 2021-04-29-core). In a first analysis, a PPI search was conducted using the 19 significantly up-regulated and spliceosome-related gene products (Table 1: genes of pathway HSA-72163) to determine the top 20 related genes. For the second analysis, the set of all significantly up-regulated proteins (Supplementary Table S1) was used without allowing inclusion of related genes.

**Table 1:** Specimen characteristics and results of pathological diagnosis of EMR specimen at different phases of the study and parts of the specimen

Specimen ID	Sex	Age	Diagnosis of patient	Diagnosis EMR, FF half	Diagnosis EMR, FFPE half	Category for statistics
ER081	M	67.0	EAC	EAC	LGD	Dysplasia/EAC
ER084	M	67.8	EAC	HGD	LGD	Dysplasia/EAC
ER086	M	62.1	EAC	LGD	LGD	Dysplasia/EAC
ER090	M	66.8	EAC	LGD	EAC	Dysplasia/EAC
ER096	M	51.6	EAC	EAC	EAC	Dysplasia/EAC
ER097	F	67.6	EAC	HGD	EAC	Dysplasia/EAC
ER102	M	67.0	EAC	LGD	n.a	Dysplasia/EAC
ER108	M	67.0	EAC	EAC	EAC	Dysplasia/EAC
ER082	M	84.7	HGD	HGD	HGD	Dysplasia/EAC
ER093	M	53.2	HGD	NDBE	n.a	Dysplasia/EAC
ER094	M	71.1	HGD	LGD	HGD	Dysplasia/EAC
ER103	M	65.0	HGD	LGD	LGD	Dysplasia/EAC
ER106	M	82.0	HGD	LGD	LGD	Dysplasia/EAC
ER083	M	71.7	NDBE	NDBE	n.a	NDBE
ER085	M	54.0	NDBE	NDBE	n.a	NDBE
ER087	M	62.2	NDBE	NDBE	n.a	NDBE
ER088	M	69.3	NDBE	NDBE	n.a	NDBE
ER089	M	80.8	NDBE	NDBE	n.a	NDBE
ER095	M	74.2	NDBE	NDBE	n.a	NDBE
ER098	M	62.2	NDBE	NDBE	n.a	NDBE
ER099	M	60.6	NDBE	NDBE	n.a	NDBE
ER104	M	58.6	NDBE	NDBE	n.a	NDBE
ER105	M	59.7	NDBE	NDBE	n.a	NDBE

Sex: *F* female, *M* male; age: age at day the sample was resected; diagnosis of patient: stage on the basis of worst pathological diagnosis; *diagnosis EMR, FF half* stage on the basis of fresh-frozen half of EMR specimen, *diagnosis EMR, FFPE half*: stage on the basis of formalin-fixed paraffin-embedded half of EMR specimen

### IHC validation

To evaluate the results of the discovery proteomics study, we performed IHC in a set of 23 formalin-fixed and paraffin-embedded (FFPE) tissue samples obtained by EMR with antibodies specific for MSH6 (1:100 diluted; AC-0047EUA, Epitomics) and XPO5 (1:400 diluted; HPA018402, Atlas Antibodies). A tissue micro-array (TMA) with 2 mm cores was prepared for 17 EMR specimens and tissue sections from 6 additional biopsy samples were mounted individually on glass slides. Stained slides were scanned and images acquired were loaded into the digital pathology software pathXL (Philips) for review and scoring by three expert pathologists. Intensity and frequency of nuclear staining of MSH6 and XPO5 and cytoplasmic staining of XPO5 were scored, and the IHC score was computed as the sum of the products of intensity and frequency of each intensity level as follows:

$${\text{score}}\;\left( {{\text{IHC}}} \right) = \mathop \sum \limits_{{I = 0}}^{3} F \times I,$$

whereby the intensity (*I*) rated the staining intensity from 0 to 3 (0 = negative, 1 = weak, 2 = moderate, and 3 = intensive) and frequency (*F*) described the proportion of epithelial cells for each intensity level (0–3). Significances between IHC scores of NDBE and dysplastic/EAC specimen were calculated by Wilcoxon rank-sum test.

## Results

### Characteristics of the sample collection and specimen

**Fig. 1:**
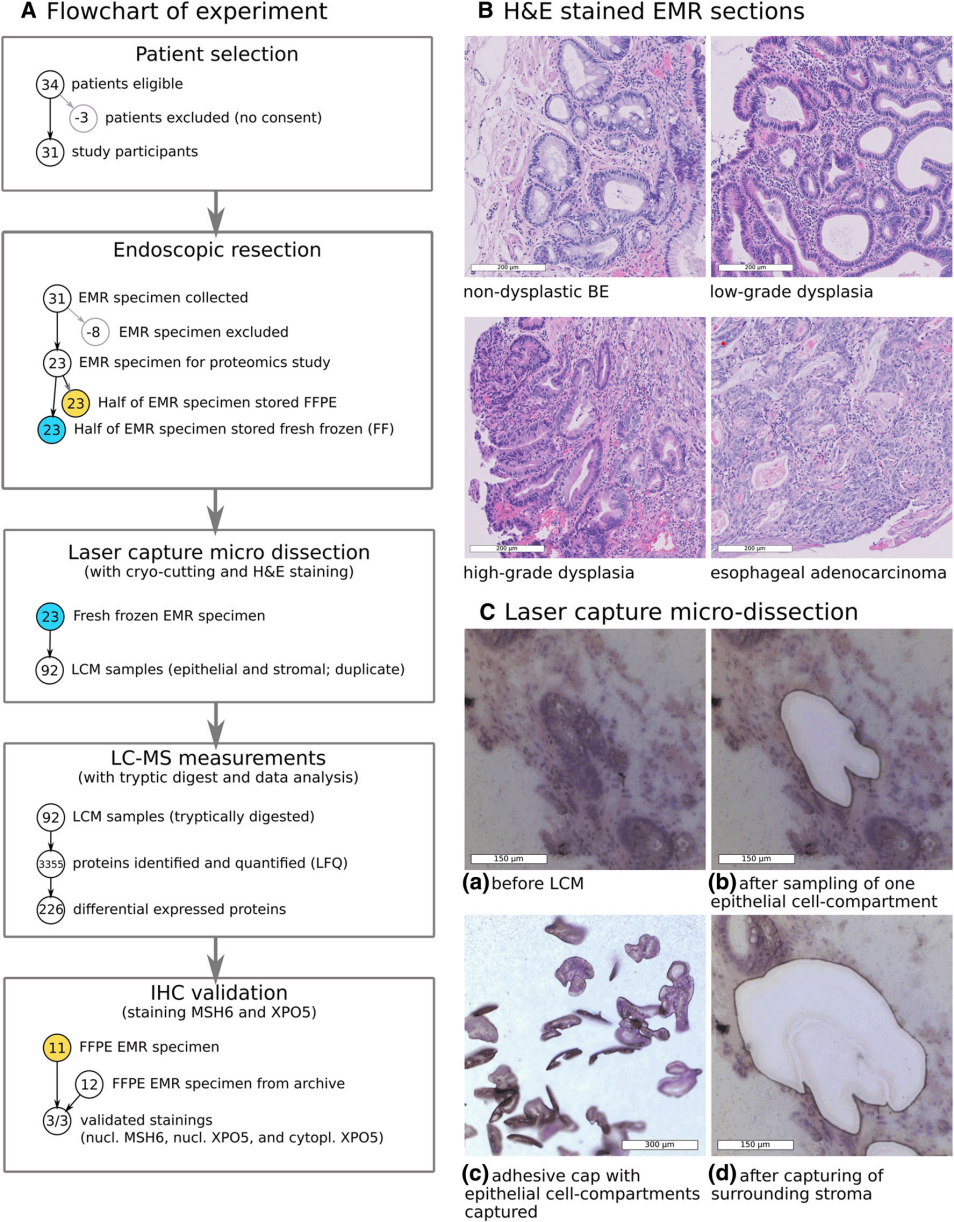
**A** Flowchart of sample collection, discovery and validation experiment. Thirty-one out of 34 initially eligible patients participated and underwent the surgical procedure, and EMR specimens of finally 23 patients could be included in the proteomics discovery experiment (samples were excluded when, e.g., the EMR was needed for clinical validation, when no EMR was taken because of submucosal growth or other endoscopic treatment). The specimens (13 dysplastic/EAC and 10 non-dysplastic) were immediately split into two halves. One half was snap-frozen (FF) for the proteomics discovery experiment, while the other half was formalin fixed and paraffin embedded (FFPE) and used for clinical evaluation. Next, in the discovery experiment, volumes of around 4.8 million µm^3^ epithelial and stromal tissue were captured by LCM of each sample in duplicate. Samples were tryptically digested, measured by LC–MS, and quantitative protein profiles were determined and compared. For the validation experiment, 11 dysplastic samples of the discovery experiment and 12 additional samples (2 dysplastic and 10 non-dysplastic) were used to score the abundances of MSH6 and XPO5 by IHC staining. **B** Representative scans of Hematoxylin–Eosin-stained EMR sections of NDBE, LGD, HGD and EAC tissue (scale bar corresponds to 200 µm). **C** Images taken during LCM showing tissue before LCM, after sampling of one epithelial compartment (micro section), all microsections of one sample collected in the adhesive cap of a sampling vial, and the tissue section after capturing of the surrounding stroma

Specimen from 11 of the initial 34 patients were excluded for the following reasons: no consent given (3), no endoscopic resection due to submucosal growth (2), small lesions for which the whole specimen was needed for clinical evaluation (3), another endoscopic treatment (radio-frequency ablation) was used instead of EMR (1), absence of dysplasia in a specimen taken from a dysplastic esophagus (1), or presence of dysplasia in a specimen taken without prior analysis of dysplasia (1). Hence, a total of 23 specimens—13 dysplasia/EAC and 10 NDBE—were used for analysis (Fig. 1). Among the patients with dysplasia/EAC, HGD was diagnosed in five cases and EAC in eight cases as the most advanced stage. Most patients were male, with only one female in the dysplastic/EAC group. Median age was comparable between groups (NDBE = 62.2 years; HGD/EAC = 67.0 years; *P* = 0.64). The median Barrett length was C3M4 for the non-dysplastic group and C3M6 for the dysplastic/EAC group.

In the course of the discovery experiment, grade of dysplasia/EAC was determined on basis of the section that was cut from the fresh-frozen EMR specimen and used for LCM. Thereby, three specimens were diagnosed as EAC, three as HGD, and six as LGD. In one sample from a patient with dysplastic BE no dysplastic tissue was found. This sample was kept in the study, but was excluded from statistical comparison between dysplastic and non-dysplastic samples. Sections of the FFPE halves of the EMR specimen used for the IHC validation experiment were reviewed and graded as well by an expert pathologist. Four EMR sections were graded as EAC, two sections as HGD and five sections as LGD (Table 1). Twelve samples of the set of FFPE halves of the initial set of EMR specimen were not available for IHC validation, and were replaced by an additional 10 non-dysplastic and two dysplastic/EAC specimens.

### Differential protein quantification

**Fig. 2:**
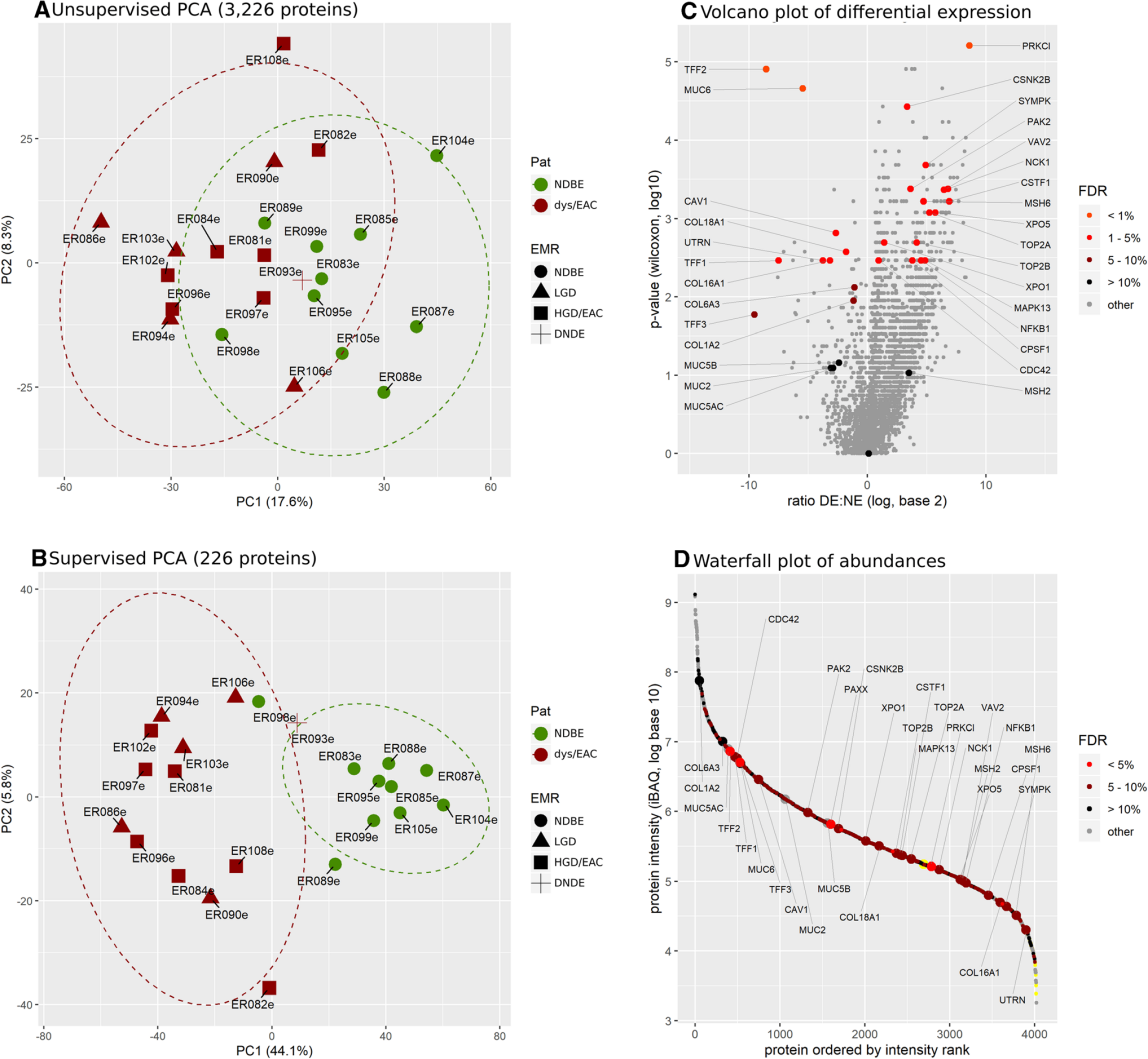
Results of protein quantification and differential quantitative analysis. Unsupervised principal component analysis (PCA) on 23 epithelial samples on the basis of 3226 quantified proteins (**A**) and supervised PCA of the set of 226 significant differentially expressed proteins (**B**). Icon colors label for dysplastic (red) and non-dysplastic (green) patients, and the icon shape refer to the stage of dysplasia assessed during the LCM experiment. Volcano plot (scatter plot of fold-change between dysplastic and non-dysplastic samples versus significance of fold-change) of differential quantitative analysis comparing dysplastic and non-dysplastic samples; colors of dots indicate the FDR of the hit (Benjamini–Hochberg correction); in total 226 proteins were found significantly up-regulated with an *FDR* < 5%, of which 209 in dysplastic samples (**C**). Scatter plot of protein abundance (iBAQ) vs intensity-based rank of protein (*waterfall plot*); red dots indicate significantly differentially expressed proteins. An abundance range of about 6 magnitudes of order is covered, and significant hits were found over almost the entire abundance range (**D**)

In epithelial samples, we quantified a total of 4059 proteins. In dysplastic/EAC we quantified 13% (*P* = 0.01) more proteins with a 48% higher total abundance (*P* = 0.04) than in non-dysplastic samples. In stromal cells, a total of 2409 proteins were quantified; the total protein abundances of dysplastic/EAC samples was not higher than that of the non-dysplastic samples. The numbers of quantified proteins and protein groups and the related responsibilities are detailed in the Supplemental Figures S1 and S2. For statistical analysis of epithelial samples, we used 3226 proteins out of these 4059 quantified proteins that were present (quantified) in at least seven samples (> 30% of samples). By unsupervised principal component analysis (PCA, Fig. 2) and unsupervised hierarchical clustering (Supplemental Figure S4A), protein profiles clustered primarily by the disease stage (non-dysplastic samples and dysplastic/EAC samples), except one LGD sample and the sample from a NDBE EMR specimen of the patient with dysplastic BE that clustered closer to non-dysplastic samples (Supplemental Figures S4A and S4B). As a result, we found 226 differentially expressed proteins (*FDR* < 5%), of which 209 were up-regulated and 17 were down-regulated in dysplastic/EAC samples (Supplemental Tables S1 and S2). In stromal samples, ratio of fold-change and significance of change between non-dysplastic and dysplastic/EAC stromal samples were calculated for 1778 proteins with a minimum occurrence of 7 samples out of the total of 2409 proteins quantified. Unsupervised hierarchical clustering and unsupervised PCA did not show formation of any distinct clusters, and no significant fold-change of protein abundance passed the *FDR* filter criteria of < 5% (Supplemental Figure S4C and Figure S5). Because of the high FDR of quantitative differences of proteins in stroma, we did not conduct further analysis on that part of the dataset. A list of all proteins identified in epithelial and stromal samples is available as supplementary data (Table S3).

### Gene set enrichment analysis

**Fig. 3:**
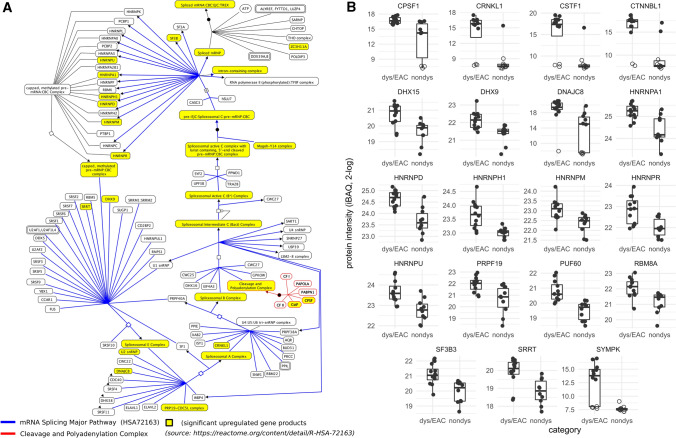
**A** Significantly up-regulated proteins in the mRNA Splicing Major Pathway (pathway source: Reactome https://reactome.org/content/detail/R-HSA-72163, pathway plot generated by Cytoscape using the ReactomeFI plugin and manually simplified and annotated; pathways with full details in Figure S6) and **B** box-plots of protein intensity grouped by disease stage of nineteen differentially expressed proteins of the mRNA Splicing Major Pathway

Gene set enrichment was carried out by querying a protein-interaction network on the basis of the 226 differential abundant proteins, further clustered by the functional interaction subnetworks which were subjected to gene set enrichment analysis using all quantified proteins (*N* = 3226) as a background reference set. We found 12 *Reactome* pathways and 6 KEGG pathways that passed the filter criteria of 5% FDR, at least five matching proteins and a minimum 5% pathway coverage (Table 2). The most significant enrichment was found for the *Reactome* pathways *mRNA Splicing—Major Pathway* (HSA-72163, 19 significant genes overlap) and the superordinated pathways *Processing of Capped Intron-Containing Pre-mRNA* (HSA-72203, 20 genes; Fig. 3 and supplemental Figure S6) and *Metabolism of RNA* (HSA-8953854, 29 genes) as well as for the KEGG pathway *Spliceosome* (map03040, 11 genes). Next, for the 19 up-regulated proteins of the splicing pathway, we determine a set of another 20 proteins based on their known and expected protein–protein interactions (*GeneMANIA* search). Interestingly, 19 of these 20 proteins were identified but were not included in the set of differentially expressed proteins because the significance of these proteins did not meet the threshold of FDR < 5%. However, when these proteins were examined individually with less stringent filtering criteria (*P* < 0.05; corresponding to an FDR < 15%), 14 of the 19 proteins passed this reduced confidence threshold (Supplementary Table S4). This accumulation of less significant proteins is nevertheless highly significant (*P* < 0.0001) and is supporting our findings. Also consistent with results of the pathway enrichment analysis, the analysis of GO term enrichment based on all up-regulated proteins also revealed that the most significantly enriched terms were associated with splicing and spliceosome-related processes (Supplementary Table S5).

**Table 2:** List of significantly enriched pathways (Reactome and KEGG) determined by String gene set enrichment analysis

Nr.	Pathway name	Src	ID	*n*	*N*	*S*	FDR	Csig%	Cid%	Genes
1	mRNA splicing—major pathway	R	HSA-72163	19	151	180	9.05e-09	12.6	83.9	CSTF1, PRPF19, SYMPK, DNAJC8, HNRNPU, SF3B3, HNRNPD, HNRNPM, DHX15, HNRNPA1, HNRNPH1, CTNNBL1, DHX9, HNRNPR, CRNKL1, PUF60, RBM8A, SRRT, CPSF1
2	Processing of capped Intron-containing Pre-mRNA	R	HSA-72203	20	179	244	9.05e-09	11.2	73.4	CSTF1, PRPF19, SYMPK, DNAJC8, HNRNPU, SF3B3, HNRNPD, HNRNPM, DHX15, HNRNPA1, HNRNPH1, CTNNBL1, DHX9, HNRNPR, CRNKL1, PUF60, ZC3H11A, RBM8A, SRRT, CPSF1
3	Metabolism of RNA	R	HSA-8953854	29	397	721	9.05e-09	7.3	55.1	CSTF1, PRPF19, WDR77, SYMPK, DNAJC8, NSUN2, HNRNPU, SF3B3, HNRNPD, NCL, HNRNPM, DHX15, HNRNPA1, HNRNPH1, CTNNBL1, DHX9, ADAR, HNRNPR, XRN2, CRNKL1, XPO1, ANP32A, PUF60, ZC3H11A, RBM8A, SUPT5H, DDX6, SRRT, CPSF1
4	T cell receptor signaling pathway	K	map04660	6	22	86	3.71e-06	27.3	25.6	MAPK13, NFKB1, PAK2, VAV2, CDC42, NCK1
5	VEGFA–VEGFR2 Pathway	R	HSA-4420097	6	37	95	0.00012	16.2	38.9	MAPK13, PAK2, CAV1, VAV2, CDC42, NCK1
6	Spliceosome	K	map03040	11	103	122	0.00014	10.7	84.4	PRPF19, HNRNPU, TCERG1, SF3B3, HNRNPM, DHX15, HNRNPA1, CTNNBL1, CRNKL1, PUF60, RBM8A
7	Proteoglycans in cancer	K	map05205	6	65	165	0.00057	9.2	39.4	MAPK13, STAT3, ARHGEF1, CAV1, VAV2, CDC42
8	TCR signaling	R	HSA-202403	6	64	126	0.00078	9.4	50.8	NFKB1, PAK2, PSMF1, PSMB10, NCK1, PSMB3
9	Fc epsilon receptor (FCERI) signaling	R	HSA-2454202	6	63	210	0.00078	9.5	30.0	NFKB1, PAK2, PSMF1, PSMB10, VAV2, PSMB3
10	Signaling by Interleukins	R	HSA-449147	8	142	452	0.00078	5.6	31.4	NFKB1, STAT3, PAK2, PSMF1, PSMB10, LCP1, CDC42, PSMB3
11	Leukocyte transendothelial migration	K	map04670	5	43	75	0.00081	11.6	57.3	MAPK13, F11R, VAV2, MLLT4, CDC42
12	Rap1 signaling pathway	K	map04015	5	46	162	0.00083	10.9	28.4	MAPK13, PRKCI, VAV2, MLLT4, CDC42
13	Tight junction	K	map04530	5	62	101	0.0018	8.1	61.4	CGN, PRKCI, F11R, MLLT4, CDC42
14	MAPK6/MAPK4 signaling	R	HSA-5687128	5	55	94	0.0026	9.1	58.5	PAK2, PSMF1, PSMB10, CDC42, PSMB3
15	Apoptosis	R	HSA-109581	6	99	179	0.0035	6.1	55.3	STAT3, PAK2, PSMF1, PSMB10, DBNL, PSMB3
16	Interleukin-1 family signaling	R	HSA-446652	5	64	139	0.0035	7.8	46.0	NFKB1, STAT3, PSMF1, PSMB10, PSMB3
17	Signaling by the B Cell Receptor	R	HSA-983705	5	60	175	0.0035	8.3	34.3	NFKB1, PSMF1, PSMB10, NCK1, PSMB3
18	C-type lectin receptors (CLRs)	R	HSA-5621481	5	71	144	0.0037	7.0	49.3	NFKB1, PAK2, PSMF1, PSMB10, PSMB3

Source = *Reactome* (R) or KEGG (K); *n* = number of significantly differentially expressed genes matching to the pathway; *N* = number of pathway-related genes products identified (used as background set); *S* size of pathway in terms of total number of genes linked to pathway (*Reactome* or KEGG). *FDR* false discovery rate of enrichment, *Csig%* coverage of identified set of pathway genes by significantly expressed genes (*n*/*N*), *Cid%* coverage of all pathway genes by identified genes (*N*/*S*)

### Validation by immunohistochemistry

**Fig. 4:**
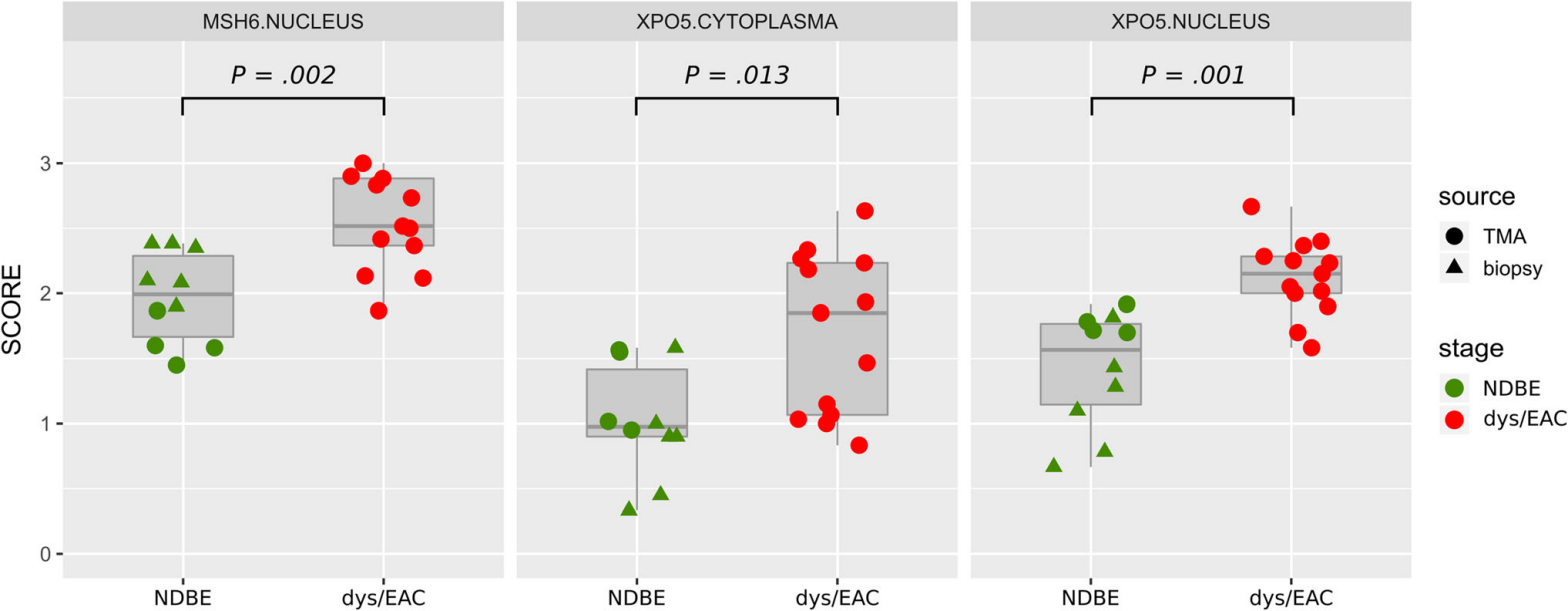
Results of technical IHC validation. IHC scores of nuclear MSH6, nuclear XPO5 and cytoplasmic XPO5 in esophageal tissue from patients diagnosed for NDBE (non-dysplastic) and dysplasia/EAC. IHC scores are the mean scores of all three reviewers; see supplemental Fig. 8 for individual scores. Significant differences (determined by Wilcoxon rank-sum test) were found in all three cases (nuclear MSH6: NDBE = 2.0 vs dys/EAC = 2.6; *P* = .016; cytoplasmic XPO5: 1.0 vs 1.8; *P* = .046; nuclear XPO5: 1.6 vs 2.0; *P* = .010, respectively) and confirmed the results of the proteomics discovery study. Icon shape indicates whether specimens were TMA cores or whole biopsies

**Fig. 5:**
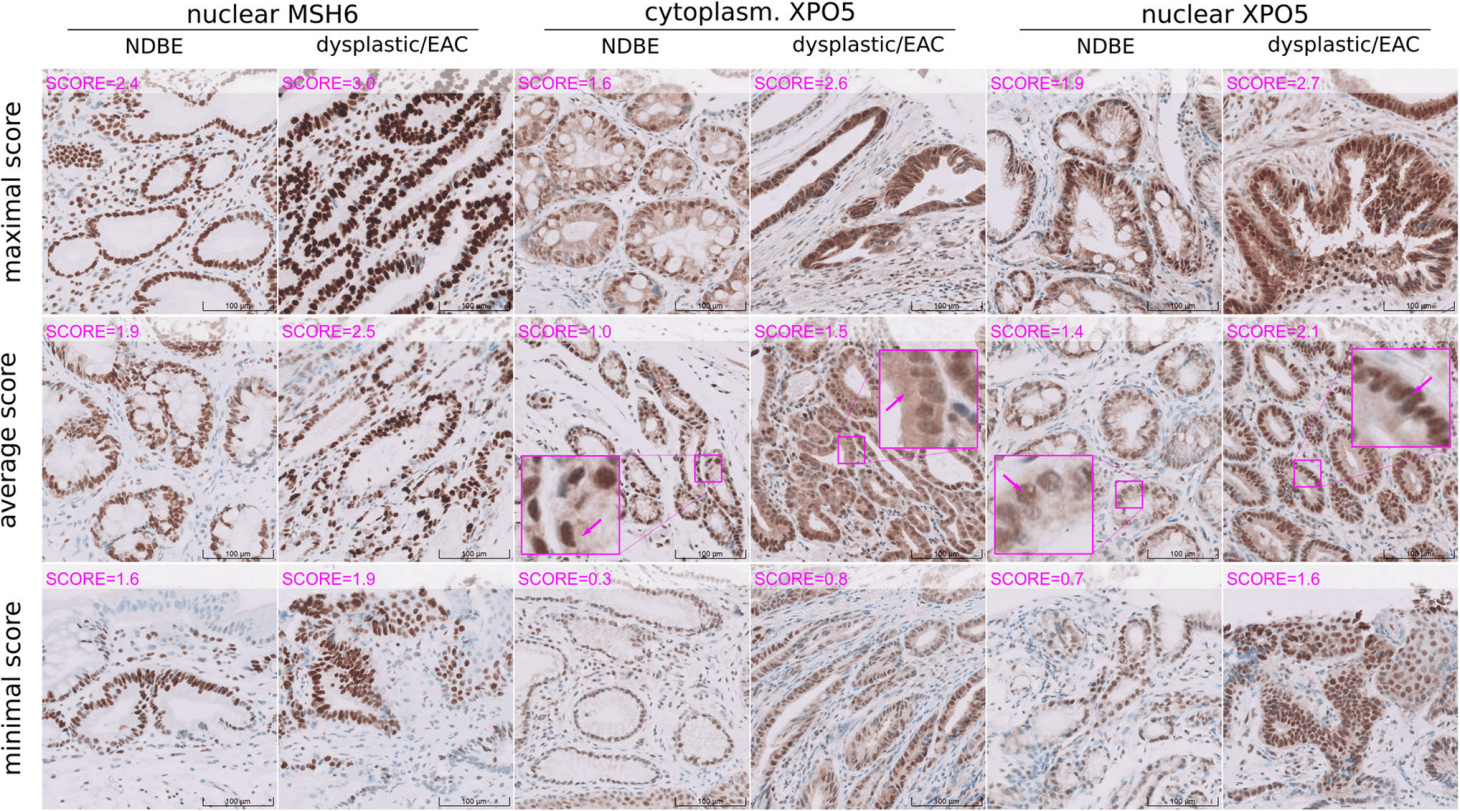
IHC staining. IHC of non-dysplastic (non-dys.) and dysplastic/EAC (dys.) samples stained for MSH6 and XPO5. Tissues were scored for nuclear MSH6, nuclear XPO5 and cytoplasmic XPO5. The sample with, respectively, the highest, middle and lowest IHC score (mean value of three reviews) is shown in the various panels. Close-ups indicating cytoplasmic and nuclear XPO5 staining (arrows). (20× magnification, scale bar corresponds to 100 µm)

For technical orthogonal validation of the discovery study, we performed an IHC staining for MSH6 (discovery study: *FDR* = 0.03, ^2^log fold-change = 4.72 up-regulated; Supplemental Table S1) and XPO5 (*FDR* = 0.03, ^2^log fold-change = 5.71 up-regulated, Supplemental Table S1) in a set of 23 FFPE samples. Staining of nuclear MSH6, nuclear XPO5 and cytoplasmic XPO5 was present in all NDBE samples (median IHC scores: nuclear MSH6 = 2.0, nuclear XPO5 = 1.6, and cytoplasmic XPO5 = 1.0) and was increased about 0.44–0.85 score points in dysplastic/EAC samples (nuclear MSH6 = 2.5, nuclear XPO5: 2.2, and cytoplasmic XPO5 = 1.9; Figs. 4 and 5; Supplemental Figure S7). Overall, the increase of IHC staining was significant when mean scores of all three pathologists were used, but also, with one exception (cytoplasmic XPO5 by one reviewer, *P* = 0.058), on the basis of the three individual reviews (Supplemental Figure S8A). Moderate to mainly strong correlations were found between the reviewers, with correlation coefficients ranging from 0.66 to 0.91 for the final review (Supplemental Figure S8B).

## Discussion

Tissue samples with different pathologic grades of the progression sequence from non-dysplastic Barrett’s epithelium, dysplasia and ultimately Barrett’s adenocarcinoma are characterized by high heterogeneity, and apparently non-dysplastic cells can be found in close spatial proximity to dysplastic and cancerous cells. In an earlier study, we used biopsies taken from non-dysplastic tissue to successfully analyze the proteomes of epithelial and surrounding stromal cells [34]. In the present study, however, using biopsies from dysplastic/EAC tissue did not prove to be a viable option for this analysis, because in the majority of cases not enough cells of the selected type and disease stage could be found. Moreover, making a clear histopathological diagnosis on the basis of a single fresh-frozen biopsy was often not possible. Therefore, for the sake of pathohistological confidence, we used fresh-frozen EMR specimen obtained by ER-cap resection as sample type to obtain biological specimen with high biological and clinical fidelity. As a consequence, sample sizes were small, but on the other hand, fewer samples had to be excluded for the reason of insufficient tissue with the targeted cell type or uncertain stage of dysplasia. Nevertheless, we still found different stages of dysplasia/EAC between different specimens of an individual but also within the same specimens, and staging coincided just partially between samples. This heterogeneity of dysplastic tissue in samples that are spatially very close to each other is probably a substantial factor for the common disagreement on grading of dysplasia reported in literature [49–52]. For the above reasons, during the statistical analysis, we were constrained to categorize solely NDBE and dysplastic/EAC tissue, without further differentiating between the grades of dysplasia.

When comparing the protein abundances between non-dysplastic and dysplastic/EAC samples, we found more different protein identifications and a higher total protein abundance in dysplastic/EAC epithelial tissue; interestingly, this observation did not hold for stromal tissue. Because same volumes of epithelial tissue were collected for both non-dysplastic and dysplastic/EAC cells, higher protein abundance is likely to be a result of the higher density of the dysplastic epithelial compartment, probably due to the absence of goblet cells and abnormal cellular organization such as atypic and crowded nuclei, and irregular compacted growth. Because for statistically analyses normalized abundances were used that corrected for variation due to overall differences, the higher number of differentially up-regulated proteins could not be exclusively explained by an overall difference of total protein abundance. The increased number and abundance of proteins are therefore probably mainly related to the higher heterogeneity of dysplastic/EAC tissue. An overall up-regulation in number of proteins was also shown in earlier studies that used LCM sampling to investigate malignant epithelial cells in breast cancer [53] and cervix carcinoma [54]. The up-regulation in these two studies as well might be explained by the possibility of a higher density of tumor cells.

In the present study, up-regulation of proteins in dysplastic samples did not apply uniformly to all types of proteins. Secreted mucins and mucin-associated trefoil factors had lower abundance in dysplastic/EAC tissue compared to non-dysplastic tissue. This group of proteins has characteristic expression patterns that in general decline during the progression from BE into EAC. The group includes MUC2, characteristically secreted by goblet cells; gastric MUC5AC, expressed at the surface epithelium and the submucosal glands; and MUC6 and MUC5B, found inside the glands [55–57]. Associated to mucins, too, is the family of trefoil factors, including the gastric tumor suppressors TFF1 and TTF2, which are co-localized with MUC5AC and MUC6, respectively; and TFF3, which is typically not secreted by gastric mucosa but, like MUC2, by goblet cells. Trefoil factors are essential in mucosal protection and repair, and decreased expression is associated with increased risk of dysplastic progression [58–60]. The presence of TFF3 in samples taken by *Cytosponges* [61] further allows specific and sensitive diagnosis of BE [62, 63]. Our results are consistent with the expected expression profile, because we found MUC6, TFF1 and TFF2 significantly down-regulated in dysplastic samples. In contrast, significant abundance differences between dysplastic and non-dysplastic samples were not found for MUC2, MUC5AC, MUC5B and TFF3. The probable reason for this latter finding is that LGD samples, from the group of dysplastic samples, had abundancies comparable to those of the non-dysplastic group. These abundance patterns of mucins and TFFs reflect the origin of secretion [59]. TFF2 and MUC6, secreted from mucous neck cells of fundic glands, show reduced expression already in LGD tissue. MUC5AC and TFF1, which are expressed in gastric mucosa cells, were similarly expressed in LGD and non-dysplastic tissue. The extent of expressions of MUC2 and TFF3, which are expressed by goblet cells, in LGD samples was between that of non-dysplastic and dysplastic/EAC tissue. However, the detection of mucins and TFFs in dysplastic/EAC samples indicates moreover that the samples collected were heterogeneous in terms of dysplasia and EAC. Collagens as well had in general a lower expression in dysplastic/EAC epithelium. Still, two of these, the endostatin precursor and angiogenesis inhibitor COL18A1 [64] and COL16A1, were significantly lower expressed in dysplastic/EAC tissue. This latter finding may be related to the proportional reduction of extra-cellular matrix surrounding epithelial cells in response to the dysplastic growth of the epithelial compartment.

A group of up-regulated proteins—PAXX, TOP2A, TOP2B, and MSH6—are involved in the stimulation of cellular response to DNA damage. PAXX is executing ligation in damage repair as response to double-strand breakage (DSB), mediated by TOP2A [65, 66]. There is further evidence that mismatch repair (MMR) genes are involved in DSB repair [66, 67] and that MSH6 regulates NHEJ activity by interaction with Ku70 [68]. Up-regulation of MSH6 and other MMR genes, such as MSH2 and MLH, have also been reported in various types of cancer [67, 69]. Mutations of MMR genes causes micro-satellite instability, which in turn leads to increased mutation rates that can ultimately lead to cancer. Still, microsatellite instability is less common in BE-associated EAC [70, 71]. In the present study, the elevated levels of MSH6 determined in the discovery experiment were confirmed by significantly increased MSH6 IHC staining in dysplasia/EAC.

Exportin-5 (XPO5) transports micro-RNAs and proteins from the nucleus to the cytoplasm [72]. MicroRNAs are small non-coding RNAs that regulate gene expression by binding to mRNA during translation, which process is frequently dysregulated in cancer [73]. In normal, dysplastic and cancerous Barrett’s epithelium, discriminating micro-RNA signatures have been found for different stages of dysplasia of esophageal tissue [74]. In colorectal cancer, elevated expression of XPO5 is primarily found in the nucleus and correlates with advanced disease stage and poor prognosis. XPO5 has an oncogenic role because its down-regulation reduces the invasive capacities and cell proliferation [75]. In prostate carcinoma, a DNA micro-array analysis revealed that XPO5 was 1.6-fold up-regulated [76]. Another exportin, XPO1, showed distinct nucleic and cytoplasmic staining patterns associated to the Gleason score [77]. In the present study, we validated the elevated expression of XPO5 in dysplastic/EAC tissue by IHC staining. This revealed a significant increase in cytoplasmic and nuclear XPO5 staining in dysplastic/EAC tissue.

The most significantly up-regulated protein in our study, PRKCI, is a known genetic driver and genomic EAC biomarker [26, 79]. PRKCI is an oncogene that shows increased copy numbers in invasive tumors and has a locus in a commonly amplified region due to 9p loss of heterozygosity during progression of BE to EAC [78, 79]. CSNK2A and CSNK2B, which both were found significantly up-regulated, are subunits of the protein kinase CK2 (CSNK2), which has been associated with various cancer types, such as breast, lung, colon, and prostate cancer. CSNK2 is an emerging candidate for targeted therapy [80]. Deregulation of the regulatory subunits is suggested to promote various cancer types, and are considered potential biomarkers and therapeutic targets [81]. In a recent study, Xiao and co-workers found that CSNK2B attenuates the inhibition of NF-κB in hepatocellular carcinoma [82].

By gene set enrichment analysis, we examined the data to identify pathways potentially underlying the set of differentially expressed gene products. On the basis of the significant up-regulation of 19 proteins, the most significantly enriched pathways identified were the *Processing of Capped Intron-Containing Pre-mRNA* pathway and the sub-pathway mRNA Major Splicing, which both are part of the RNA metabolism super-pathway. These pathways stood out for their highly significant enrichment (FDR = 9e−9) and differed distinctly in this respect from other pathways (e.g., 4th pathway, *T cell receptor signaling*: FDR = 4e−6). Strong enrichment for spliceosome components was earlier found in a study by Francavilla and co-workers in epithelial ovarian cancer by using a mass-spectrometry-based proteomics approach [83]. A meta-analysis comparing four publicly available BE- and EAC-associated micro-array datasets, published by Nangraj et al. revealed that RNA metabolism and spliceosome are critical in the formation and development of EAC [84]. The manifold and complex associations of spliceosome and cancer have been reviewed in detail by Srebrow et al. and El Marabti et al. [85, 86] What it basically comes down to is that due to mutations and alterations of expression levels of the splicing factors, the cancer is able to affect splicing and, thus, is potentially able to promote the selection of certain splicing variants. This observation is relevant for the pathology of cancer because, on the one hand, the functions of a protein are often related to the splicing form and, on the other hand, alternative splicing affects more than 90% of human genes [87]. Thus, changes of splicing factors can affect the splicing isoform selection—and hence processes related to cancer [85]. Dysregulation of splicing has in multiple studies been linked to cancer development, involving both oncogene and tumor suppressor activities [88]. Jiménez-Vacas et al. showed that the up-regulation of splicing factor SF3B1 is associated with the expressions of oncogenic splicing variants and the progression of prostate cancer [89]. Highly relevant in the development of EAC is the expression of different p53 protein isoforms as a result of different TP53 splicing forms [90]. Equally relevant is that MYC, an EAC driver gene [26], regulates the splicing of selected genes via the activation of alternative splicing factors or components of the core spliceosome [91, 92]. Spliceosome core components have been suggested as potential therapeutic target in various types of cancer, such as lung, breast, ovarian and prostate cancer [89, 93]. In the present study, we found the highest up-regulation within the spliceosome for the *cleavage and polyadenylation complex*, due to elevated expression of CPSF1, CSTF1 and SYMPK, potentially leading to deregulation of alternative polyadenylation (APA), which further yields mRNA 3′ untranslated region (UTRs) isoforms with modified characteristics including oncogenic activities [94]. For example, an SNP in the 3′ UTR of TP53 that is transcribed as a consequence of APA (lengthening) forms a risk factor for different types of cancer, such as prostate cancer, glioma, and colorectal adenoma [94, 95]. The regulation of proliferation marker Ki-67, which we found up-regulated, is as well mediated by APA in breast cancer [96].

IHC staining of nuclear MSH6 and nuclear and cytoplasmic XPO5 was observed in all samples. This observation partly reflects the results of the MS-based discovery study, in which XPO5 and MSH6 were found almost exclusively in dysplastic samples—and not in non-dysplastic samples. The discrepancy between absence in the discovery experiment and presence in the validation experiment might be primarily due to limited sensitivity of mass spectrometric detection, but could also be related to limited selectivity of the antibodies used. Nevertheless, the latter consideration seems unlikely, especially in the case of MSH6, because we used clinically validated antibodies. Hence, the relatively high fold-change ratios of the discovery study relative to those found during IHC validation are likely a result of zero imputation during statistical analysis. It must be noted, however, that the significance determined was not affected by zero imputation because a non-parametric test was applied. Furthermore, a linear quantitative relationship need not necessarily be assumed between the IHC score and the actual protein concentration of the tissue or the protein concentration determined in the discovery phase by mass spectrometry. In summary, both proteins had significantly higher IHC scores in dysplastic/EAC tissue, and thus positively validated the result of the discovery experiment.

In summary, this study provides insights in the alteration of epithelial proteomes during progression from NDBE into EAC. We determined a set of differentially expressed proteins that overall are up-regulated in dysplasia/EAC and point to increased activity of DNA mismatch repair, micro-RNA transport and RNA splicing. We showed increased immunostaining of MSH6 and XPO5 to confirm these findings. The proteomic finding of associations of spliceosome and polyadenylation activity with dysplastic progression of BE confirms recent novel findings, and extends the current knowledge of Barrett’s carcinogenesis.

## Supplementary Information

Below is the link to the electronic supplementary material.

Supplementary file1 (XLSX 347 KB)

Supplementary file2 (PDF 4186 KB)

## Abbreviations

AGC: Automatic gain control
APA: Alternative polyadenylation
BE: Barrett’s esophagus
DSB: Double-strand breakage
EAC: Esophageal adenocarcinoma
ELISA: Enzyme-linked immunosorbent assay
EMR: Endoscopic mucosa resection
ER-cap: Endoscopic resection cap technique
ESI: Electrospray ionization
FDR: False discovery rate
FFPE: Formalin fixed and paraffin embedded
GERD: Gastroesophageal reflux disease
GO: Gene ontology
HE: Hematoxylin and eosin
HGD: High-grade dysplasia
iBAQ: Intensity-based absolute quantification
IHC: Immunohistochemistry
LC–MS: Liquid chromatography coupled to mass spectrometry
LC: Liquid chromatography
LCM: Laser capture microdissection
LFQ: Label-free quantification
LGD: Low-grade dysplasia
MALDI: Matrix-assisted laser desorption/ionization
MMR: Mismatch repair genes
MS/MS: Tandem (or fragment) mass spectrum
NDBE: Non-dysplastic Barrett’s epithelium
NHEJ: Non-homologous end joining
SNP: Single-nucleotide polymorphism
TMA: Tissue micro-array
u: Atomic mass unit
UTR: Untranslated region
