# Supplementary material for: Alteration of protein expression and spliceosome pathway activity during Barrett’s carcinogenesis
Source: J Gastroenterol. 2021 Jul 5;56(9):791–807. doi: 10.1007/s00535-021-01802-2 (PMC8370908; doi:10.1007/s00535-021-01802-2)
Supplement: Supplementary file 2 — Supplementary file2 (PDF 4186 KB) [file 535_2021_1802_MOESM2_ESM.pdf]

Alteration of protein expression and spliceosome pathway activity during Barrett's carcinogenesis

Christoph Stingl[1,*], Angela Bureo Gonzalez[2], Coşkun Güzel[1], Nadine Phoa[2], Michail Doukas[3], Gerben E. Breimer[4,5], Sybren L. Meijer[4], Jacques J.G.H.M. Bergman[2], Theo M. Luider[1]

[1]Department of Neurology, Erasmus University Medical Center, Rotterdam, the Netherlands;

[2]Department of Gastroenterology and Hepatology, Amsterdam University Medical Centers, Amsterdam, the Netherlands;

[3]Department of Pathology, Erasmus University Medical Center, Rotterdam, the Netherlands;

[4]Department of Pathology, Amsterdam University Medical Centers, Amsterdam, the Netherlands;

[5] Department of Pathology, University Medical Center Utrecht, the Netherlands (present address);

[*]corresponding author

Supplemental figures

- **Supplemental Figure S1:** Number of protein identification per LC-MS run for epithelial and stromal samples.
- **Supplemental Figure S2:** Overall quantitative differences of epithelial and stromal samples.
- **Supplemental figure S3:** Results of pathological diagnosis of EMR specimen
- **Supplemental Figure S4:** Annotated dendrograms of hierarchical cluster analysis of epithelial and stromal samples.
- **Supplemental Figure S5:** Results of differential protein quantification of stromal samples.
- **Supplemental Figure S6:** Significant upregulated proteins in Reactome pathways Processing of capped intron pre-mRNA pathway and mRNA Splicing Mayor Pathway, and all proteins of mRNA Splicing Mayor Pathway annotated in volcano plot of epithelial samples.
- **Supplemental Figure S7:** Non-dysplastic and dysplastic/EAC samples IHC stained for MSH6 and XPO5.
- **Supplemental Figure S8:** Results of technical IHC validation of three reviewers.

Supplemental Figure S1

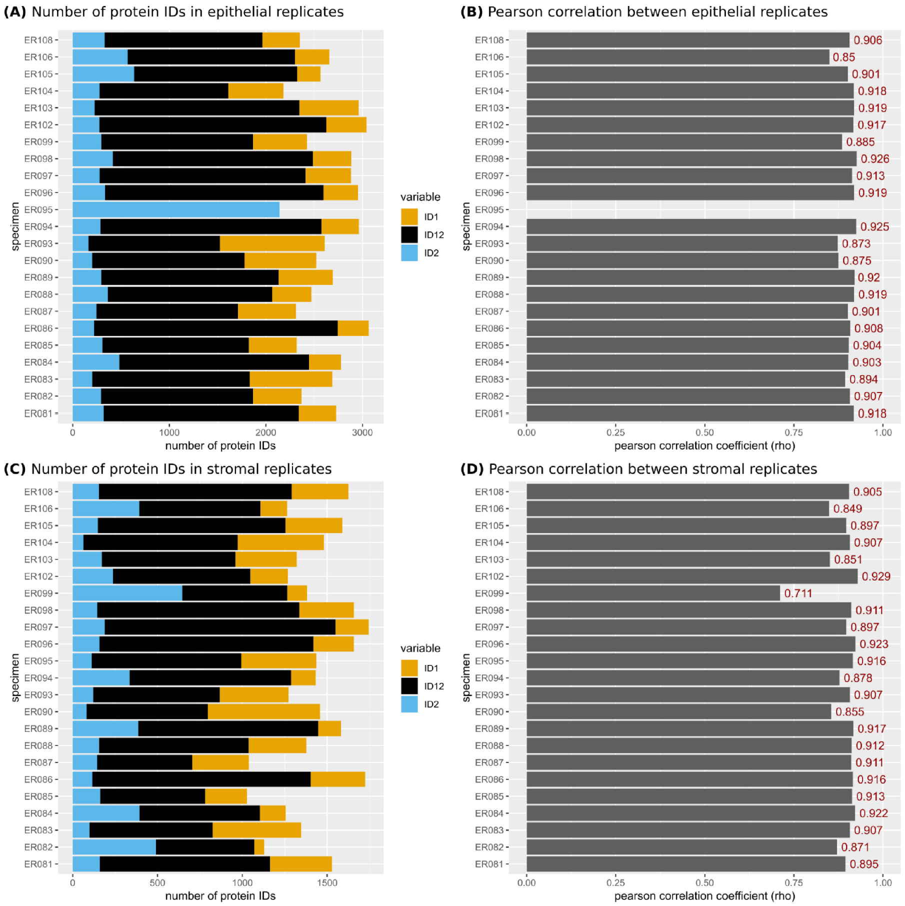

Supplemental Figure S1: Number of protein identification per LC-MS run for epithelial (top) and stromal (bottom) samples**.** Left side: Number of identified proteins exclusively found in the first replicate (ID1) and second replicate (ID2) are shown orange and blue, respectively. Total number of protein IDs per replicate measurement 1 and 2 are the sum of orange and black and the sum of blue and black, respectively. Black sections (ID12) in the bar plots show the number of protein identifications found in both replicates of one sample and were used to calculate the quantitative correlation. Right side: Correlation (Pearson) of protein abundances (label-free iBAQ values) as used for statistics (after normalization and ^{2}log based -transformation) of the two replicates per run. **Panel 1A:** In epithelial samples, we identified and quantified a total of 3,890 protein groups corresponding to 4,059 proteins, and per run we identified on average 2,242 proteins (sd = 335, RSD = 14.9%) and per sample (both replicates combined) on average 2,632 proteins (sd = 279, RSD = 10.6%); **Panel 1B:** Correlation between proteins quantified in both replicates was on average 0.90 (Pearson correlation coefficient, RSD = 2.1%); **Panel 1C:** In stromal cells a total of 2,275 protein groups corresponding to 2,409 proteins were quantified. Per runs on average 1,157 proteins (sd = 260, RSD = 22.5%) and per sample on average 1,417 proteins (sd = 205, RSD = 14.5%) were identified and quantified, respectively, **Panel 1D:** A correlation of on average 0.89 (Pearson; RSD = 5.1%) was found between the abundance of proteins in the two replicates of one sample. (Supplemental Figure S1C and 1D).

Supplemental Figure S2

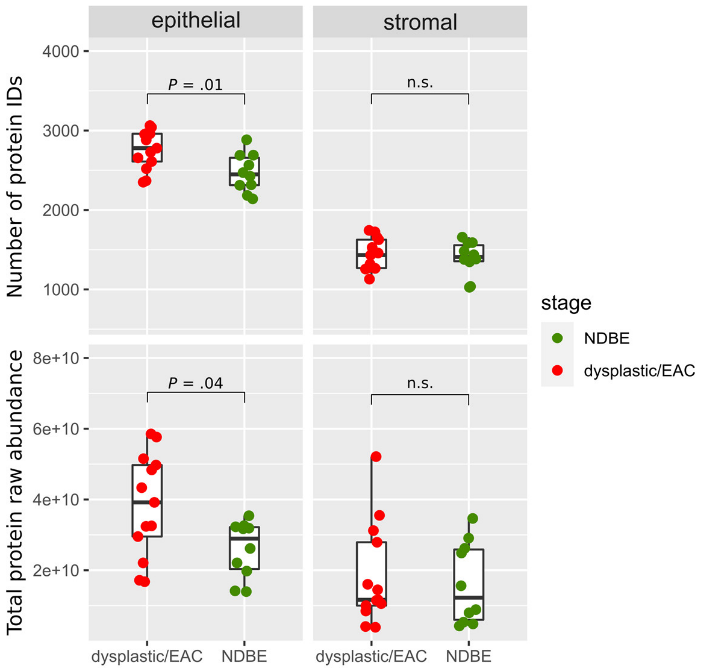

Supplemental Figure S2: Overall quantitative differences of epithelial (left) and stromal (right) samples in terms of the number of protein identification (top) and total protein raw abundance (bottom). In epithelial samples, on average 13% more proteins were found in dysplastic/EAC samples (P = .01) and total protein raw abundance was 48% higher in dysplastic/EAC samples (*P* = .04). The term total protein raw abundance refers hereby to the sum of iBAQ abundances of all quantified proteins in a sample before normalization**.** In stromal samples, the number of identifications and total protein raw abundances did not differ significantly between dysplastic/EAC and non-dysplastic samples.

Supplemental Figure S3

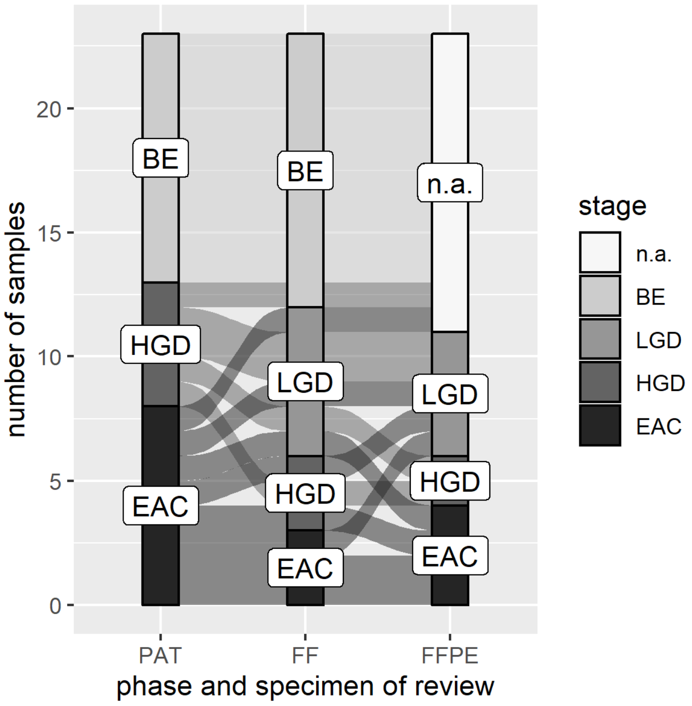

Supplemental Figure S3: Results of pathological diagnosis of EMR specimen at different phases of the study and parts of the specimen. Number of samples diagnosed for NDBE, LGD, HGD, and EAC at the initial time point of patient selection and sample collection (PAT), during pathologically review in the course of the MS proteomics discovery study on the fresh frozen half of the EMR specimen (FF) and during the IHC validation experiment using the formalin-fixed paraffin-embedded half of the EMR specimen (FFPE). FFPE half of EMR specimen from NDBE patients that were not available for the IHC validation study are labelled with 'n.a.' (not available).

Supplemental Figure S4

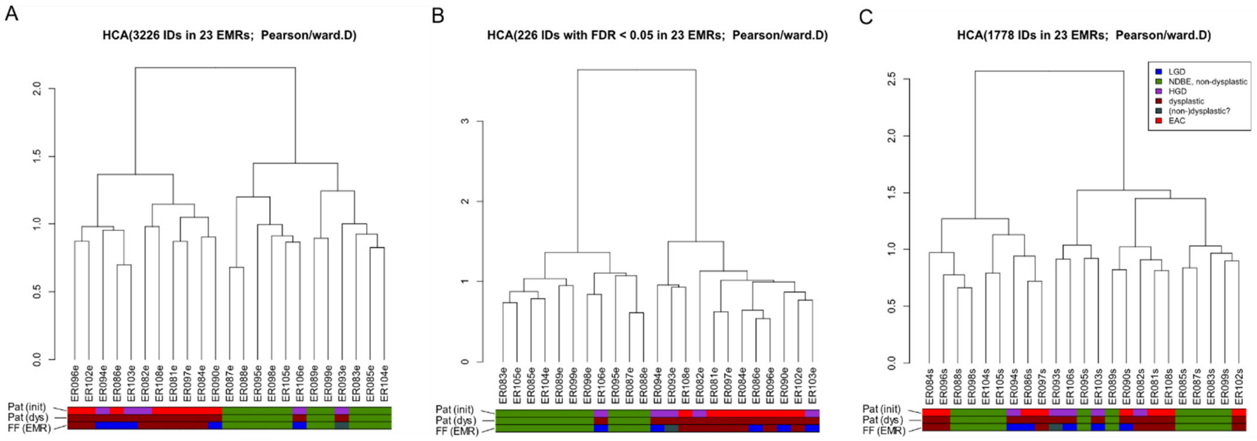

Supplemental Figure S4: Annotated dendrograms. Dendrograms were derived from hierarchical clustering on basis of Pearson correlation and Ward hierarchical clustering of three data sets: **(Panel A)** unsupervised clustering of 3,226 proteins quantified in epithelial cells; **(Panel B)**: supervised clustering of 226 proteins that were significant different expressed in dysplastic and non-dysplastic epithelial cells and **(Panel C)** unsupervised clustering of 1,778 proteins quantified in stromal cells. The color bar below the dendrogram labels the initial diagnosis of the patient (NDBE, HGD< or EAC) **(init)**, after patho-histological classification of the **(pat)**, and categorization of the samples on basis of the pathological review of the EMR section that were actually the used source material for laser capture micro-dissection and down-stream proteomics analysis **(EMR)**.

Supplemental Figure S5

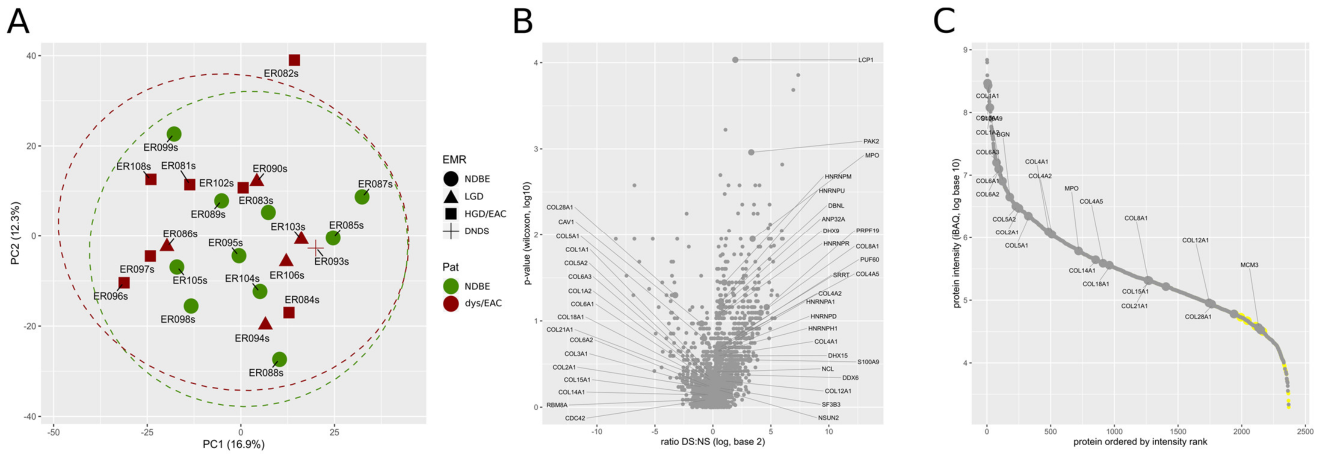

Supplemental Figure S5: Results of differential protein quantification of stromal samples. (Panel A) Unsupervised principle component analysis (PCA) on 23 stromal samples on basis of 1,778 quantified proteins. Icon colors label for dysplastic (red) and non-dysplastic (green) patients and the icon shapes refers to the stage of dysplasia assessed during the LCM experiment (NDBE: non-dysplastic BE, LGD: low-grade dysplasia, HGD/EAC: high-grade dysplasia or EAC, DNDS: NDBE specimen from HGD esophagus). **(Panel B)** Volcano plot (scatter plot of fold change between dysplastic and non-dysplastic samples versus significance of fold-change) of differential quantitative analysis comparing dysplastic and non-dysplastic samples; colors of dots indicate the FDR of the hit (Benjamini-Hochberg correction); no significant different passing FDR < 5% were found. **(Panel C)** Scatter plot of protein abundance (iBAQ) vs intensity-based rank of protein (waterfall plot).

Supplemental Figure S6

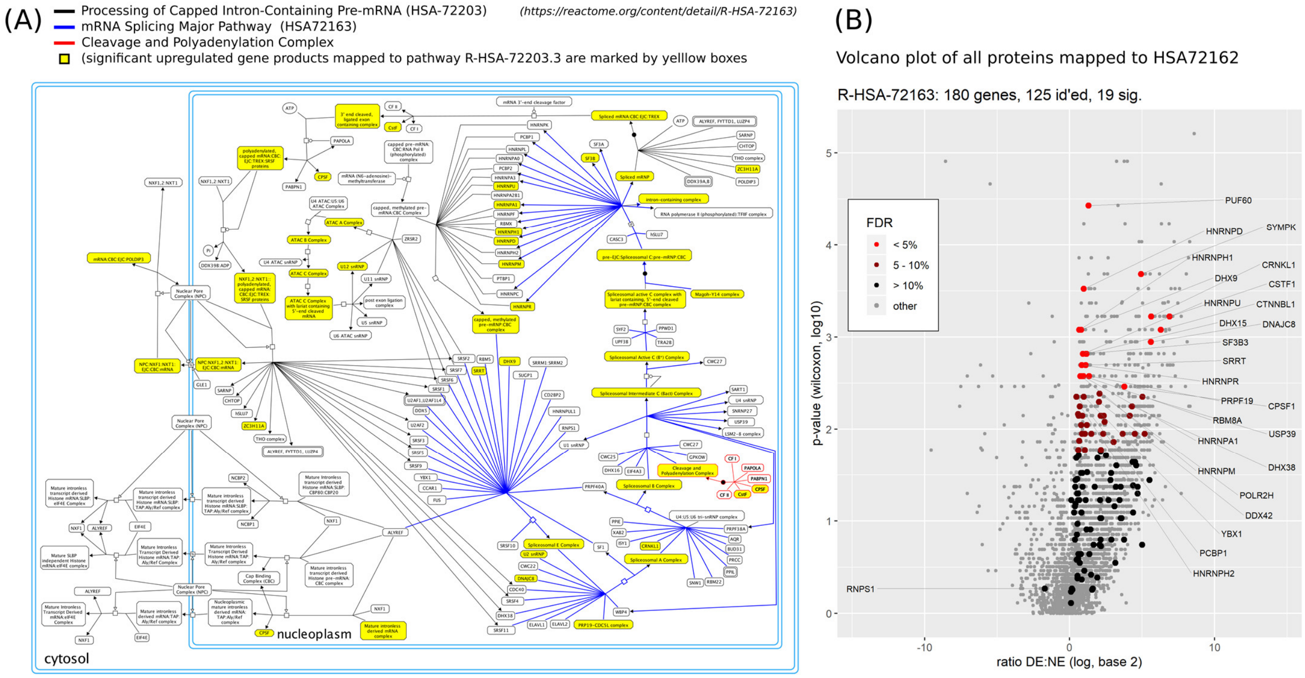

Supplemental Figure S6: *(A)* Significant upregulated proteins in *Reactome* pathways *Processing of capped intron pre-mRNA pathway (HSA-72203) and mRNA Splicing Mayor Pathway (HSA-72163)* and all (B) proteins of *mRNA Splicing Major Pathway* annotated in volcano plot of epithelial samples. (Panel A) mRNA Splicing Mayor Pathway (HSA-72163, blue edges), part of the Processing of capped intron pre-mRNA pathway (HSA-72203, entire network), was found by gene set enrichment analysis with highest confidence (FDR = 9e-9) by the overlap of 19 out of 151 genes (marked yellow). The relatively strong upregulation of CSTF1, CPSF1 and SYMPK indicated increased expression of *Cleavage and polyadenylation complex* (red edges); pathway plot was generated by Cytoscape using the ReactomeFI plugin and manually annotated. **(Panel B)** Volcano plot annotated by all proteins of *mRNA Splicing Mayor Pathway.*

Supplemental Figure S7

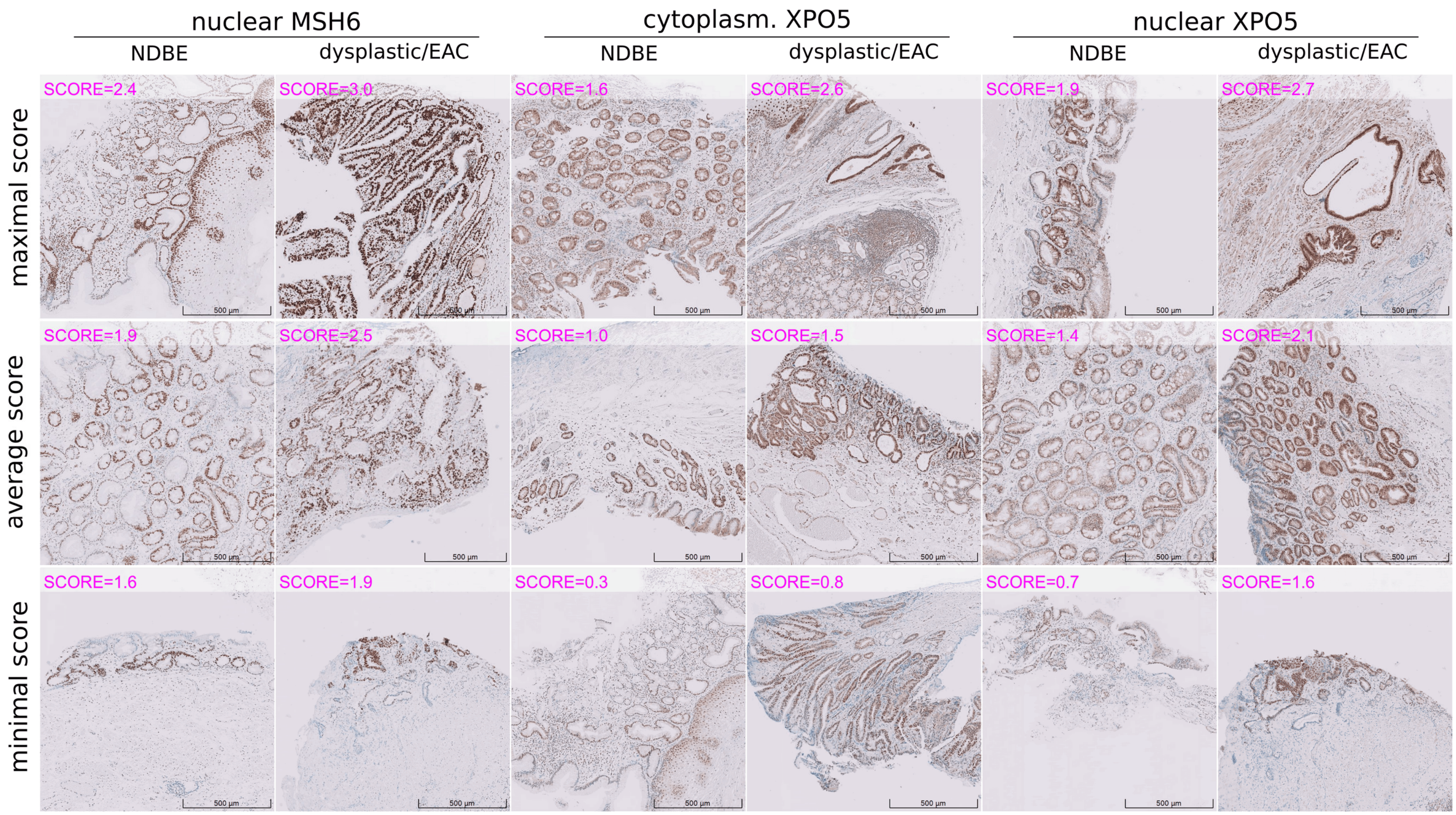

Supplemental Figure S7: Non-dysplastic and dysplastic samples IHC stained for MSH6 and XPO5, that were scored for nuclear MSH6, nuclear XPO5 and cytoplasmic XPO5. The samples with the highest, middle and lowest IHC score determined (mean value of three reviews) are shown as examples. (5x magnification, scale bar corresponds to 500 µm)

Supplemental Figure S8

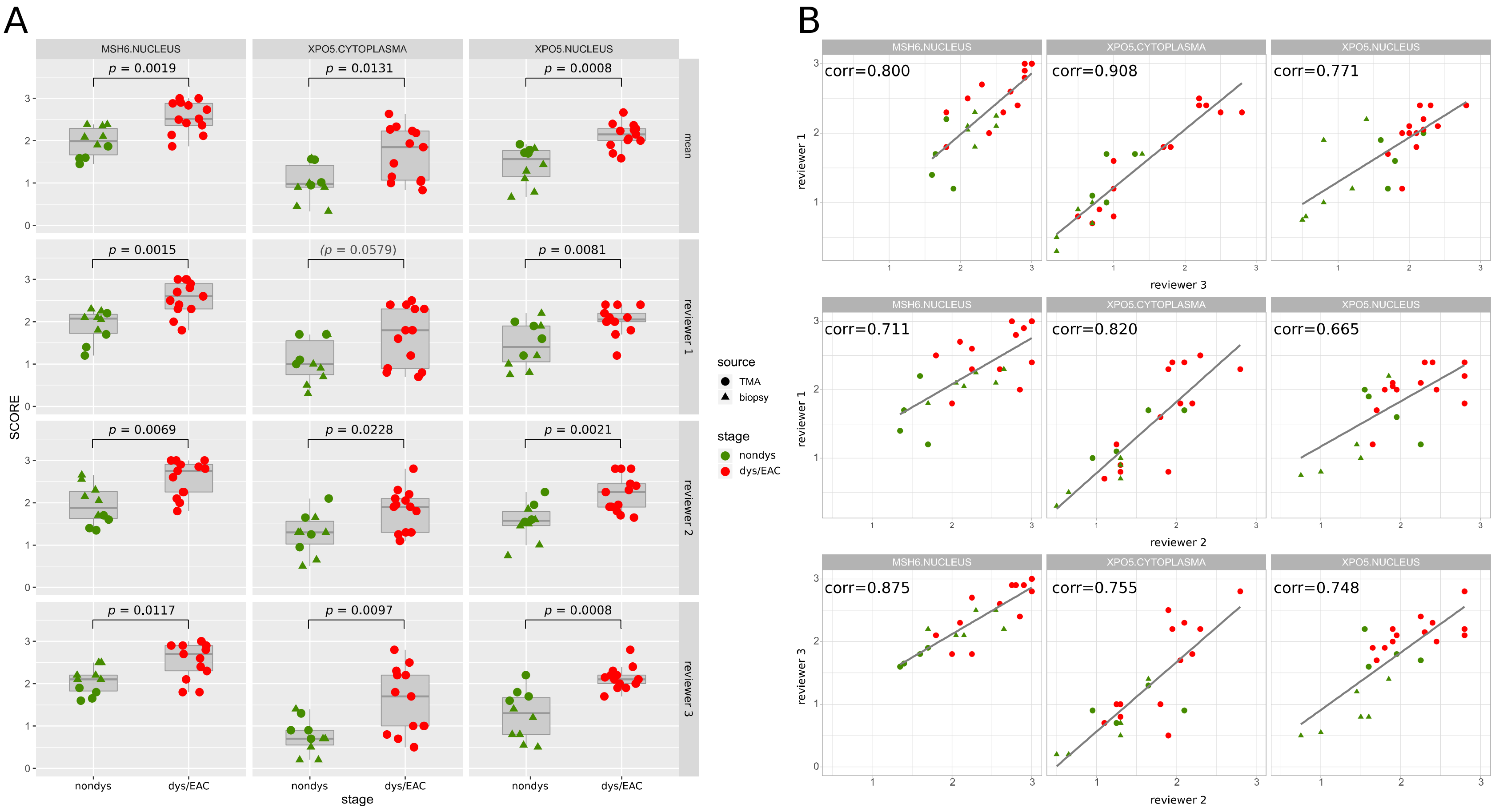

Supplemental Figure S8: (A) Results of technical IHC validation by three pathology trained reviewers. IHC scores of nuclear MSH6 and XPO5 and cytoplasmic XPO5 in non-dysplastic and dysplastic esophageal tissue determined by three reviewers. Significant differences in IHC score was found for all reviews except the assessment of cytoplasmic XPO5 by pathologist 1 (*P* = .0579); **(B) Correlation of IHC results of nuclear MSH6, nuclearXPO5 and cytoplasmic XPO5 among pathologists.** Correlations (corr) were calculated as *Spearman* rank correlation coefficient. Median correlation was 0.77 (min = 0.66, max = 0.91)
